# Supplementary material for: The expectations and acceptability of a smart nursing home model among Chinese elderly people: A mixed methods study protocol
Source: PLoS One. 2021 Aug 23;16(8):e0255865. doi: 10.1371/journal.pone.0255865 (PMC8382194; doi:10.1371/journal.pone.0255865)
Supplement: S1 Appendix — (PDF) [file pone.0255865.s001.pdf]

Appendix 1. Semi-structured interview guides (For elderly people and the family members)

Topics	Questions	Follow-up Probes
Perception of a smart nursing home	1. Can you talk about your perception of a smart nursing home?	Can you tell us more about it?<br>If Yes, Why?<br>If not, Why?<br>Can you explain it?<br>What do you mean?
	2. What do you think a smart nursing home should look like? or What kind of nursing home could be defined as a smart nursing home?	
	3. What do you think is the difference between a smart nursing home and a traditional nursing home?	
Expectation on a smart nursing home	1. What do you expect from a smart nursing home?	
	2. What are the most important features that a smart nursing home should have?	
	3. Do you ***(your senior family members)*** have any medical needs that a nursing home cannot meet your demand?	
	4. Do you think a nursing home should have the integration of medical services?	
	5. What technologies will you expect in a smart nursing home?	
	6. What else do you expect in a smart nursing home?	
Acceptability of smart technologies and a smart nursing home	1. Would you allow yourself to be admitted to a smart nursing home? ***Or Do you accept to send your senior family members into a smart nursing home?***	
	2.What technology will you accept to adopt for your ***(your senior family members)*** daily assisting living?	
	3. Who and how will you be persuaded to adopt a smart technology? ***(This question is only for elderly people)***	
	4. What will be the key reason for you to adopt a smart technology?	
