# Supplementary material for: The expectations and acceptability of a smart nursing home model among Chinese elderly people: A mixed methods study protocol
Source: PLoS One. 2021 Aug 23;16(8):e0255865. doi: 10.1371/journal.pone.0255865 (PMC8382194; doi:10.1371/journal.pone.0255865)
Supplement: Attachment — Submitted filename: Response to Reviewers-PONE-D-20-23914.docx [file pone.0255865.s002.docx]

| To PLOS ONE  Replies to comments to the authors  Manuscript ID: PONE-D-20-23914  Title of Manuscript: The Chinese elderly people’s expectation s and acceptability to a smart nursing home model: A mixed methods study protocol  Replies to the comments of the reviewers  Dear Reviewers,  We would like to thank you for taking the time to review our manuscript and we thank you for your valuable comments. We have revised the manuscript. Below, please find our replies to your comments. | |
| --- | --- |
| **Reviewer(s)' General Comments to the Author:** | |
| **Comment 1** | **Clarify the study motivation discussing the original contributions in comparison to existing works.** |
| Reply | We have revised the introduction accordingly. Please see the last paragraph of introduction on page 6: ‘These technologies include telemedicine…. are imperative for a successful smart nursing home model.’ |
| **Comment 2** | **Clarify the objectives of the study and make explicit the research questions and the research hypotheses.** |
| Reply | We have revised accordingly. Please see the section of ‘Theoretical framework’ and ‘Study aims and objectives’ on page 7 and 8. |
| **Comment 3** | **Revise and clarify theoretical framework section: for each mentioned construct provide definition, indicators, measurement tools, etc.** |
| Reply | We have revised the theoretical framework and figure 1. |
| **Comment 4** | **Clarify how data collection tools are derived from research framework**. |
| Reply | This study is guided by the smart technology adoption behaviours of elder consumers theoretical model proposed by Golant (2017), the expectation of Chinese elderly people and unmet needs to a smart nursing home will be explored and the theory will be explored in Phase I through qualitative study and adapted as necessary based on the findings. The emerging codes will be analyzed thematically and according to the framework if they are coherent. The codes will become variables, themes will become scales, and the quotations from raw data will become questionnaire items. We plan to use a 5-point Likert scale to measure the constructs of expectation (unmet needs appraisal, ability to understand smart technology) and acceptability to a smart nursing home. Expectation may be conceptualized in unmet needs appraisal, ability to understand smart technology, resilience (self-efficacy and openness) to new information about the technology understanding/use. Acceptability will be conceptualized in readiness to adopt the smart technology and a smart nursing home. Please see our revision in line 319, paragraph 2, page 13 and line 340, paragraph 2, page 14. |
| **Comment 5** | **Clarify the selection criteria of the two specific cities for the purpose of the interview.** |
| Reply | Please see the revision in line 237, paragraph 2, page 10-11: ‘Every October, there are 1.64 million ‘migratory birds elderly people from 27 Chinese provinces coming to Hainan Island ...elderly people from other provinces who either buy own apartments or reside the nursing homes in Dalian’. |
| **Comment 6** | **Clarify selection criteria of subjects for the semi-structured and the in-depth interviews.** |
| Reply | The subjects for in-depth interviews and semi-structured interviews include both the elderly people and their family members. The inclusion and exclusion criteria are 1) migrants aged 60-75 years old from other parts of China (citizens) living in Hainan Island and Dalian; 2) local residents living in Hainan and Dalian; and 3) those having only one child or two children who may have difficulty in providing care for their parents. To be excluded are elderly people who are not citizens of China and those who are unwilling to be interviewed or are not able to communicate clearly due to language problems, such as dysphagia and dementia. In order to get more insights on the expectations and acceptability of smart nursing homes, the family members of the eligible elderly people will also be included in the interview. The inclusion criteria include: 1) being employed or are self-employed, 2) aged ≥30 years old, and 3) have relatives who stay in the same house with the elderly or take care of the elderly by helping them with certain chores such as cooking and cleaning the house, or assisting the elderly in daily routines. Other family members including grandchildren and children-in-law will be excluded.  Please see the ‘section of participants’, page 11. |
| **Comment 7** | **Include in the research recent Chinese Smart Home care work.** |
| Reply | We have included an additional related work: ‘In 2015, Chinese government proposed 'Internet Plus' plan to address technology innovation and initiate integrating social services to build smart homes…This brings about many challenges to the balance development, quality maintenance and sustainability of the ‘traditional’ nursing homes’. Please see line 110, page 5. We would appreciate to be informed if they are other related ‘recent Chinese Smart Home care work’’ to this study. |
| **Comment 8** | **Extend the review section.** |
| Reply | We have reconstructed the introduction. |
| **Comment 9** | **Revise the abstract.** |
| Reply | We have revised accordingly. |
| **Comment 10** | **Revise the English language.** |
| Reply | We have acquired a proofreading service for this revised manuscript. |
| **Comment 11** | **Improve the manuscript readability e. g. simplifying complex phrases.** |
| Reply | We have revised and submitted the manuscript to a professional proofread. |

| **Comments of Reviewer 1:**  Reviewer #1: The authors focus on a well-investigated topic in current literatures. I do not find how the proposed work is different from the existing ones that I have mentioned below in details. My decision for this manuscript will therefore be a reject. | |
| --- | --- |
| **Comment 1** | **1. The study motivation is not clear. The authors aim to investigate the elderly people’s intention towards using smart technologies in a nursing home setting. First, the term “smart technologies” must be clarified. It is too broad, and unless a proper scope of the technology is given it will be difficult to comprehend the usage scenario. Second, from the definition of nursing home it seems as if the authors are referring to some form of an old-age home. Generally nursing homes provide critical care too with doctors and nurses being present round-the-clock. Often, they have mini operation theaters and even an ICU/HDU unit in some cases. So, elaborate on this aspect that whether the acceptance model that you propose includes critical care or focus only on the general well-being aspect of the elderly. The Introduction section is too vague and the neither the research gaps nor the research contributions are clear. Moreover, all forms of healthcare institutes have already embraced various forms of ICT technologies for their day-to-day operations. Therefore, from an elderly perspective what aspects of smart technology do you plan to focus on is unclear as there are numerous works already available in this regard.** |
| Reply | 1-1: The study questions and objectives are clearly stated on page 8. In short, this study aims to explore the expectation and acceptability of a smart nursing home among the mainland Chinese elderly people and their family members. These two constructs are defined at line 163, on page 7. ‘Expectations are desires or wants of consumers of what they feel a smart nursing home should offer. Acceptability is present when factors or services that satisfy the target users and they are willing to use or adopt the smart nursing homes’  We also realize the importance of a clearly defined smart technology and smart nursing homes before embarking on this mixed methods study. Based on the literature, we define smart technologies as ‘a new generation of information technologies such as the Internet of Things (IoT), big data, cloud computing and artificial intelligence could transform the traditional medical healthcare more efficient, convenient and personalized. In addition, the World Health Organization (WHO) (2019) associates smart technologies in healthcare with digital health including telemedicine and mobile applications for health’. Please see line 102, page 5.  We preceded this study with a scoping review (ScR) conducted by the same research team on the concepts of a smart nursing home, its aspects of technology feasibility, integration of medical services and acceptability factors. This ScR protocol has been accepted for publication in BMJ Open, and we have used some of the knowledge gained in the revision of this protocol.  1-2: The smart nursing home models in this study include the majority of the elderly people without terminal illnesses, not bed-bound or requiring critical care. These nursing homes are without ICU/HDU units. Please see ‘Study aims and objectives’ on page 8.  1-3: We have reconstructed the introduction section and elaborated the research gaps, research contributions and clarified the research questions. Please see the revised introduction section and the section of ‘Study aims and objectives’ on page 8.  1-4: We agree that many healthcare facilities have already embraced various forms of ICT technologies for their day-to-day operations. However, expectation of the target users, the elderly people and their family members themselves, of the types and extend of the ICT technologies to be incorporated in nursing homes are largely uncertain (see our earlier reply for general comment 7 on the available literature of similar works). Moreover, the elderly people and their family members acceptability of a smart nursing home is the complementary aspect of expectation to be explored and ascertained. This is equally if not more important as it would provide the realistic reasons when the prospective target users consider to utilize or not the smart nursing home. Therefore, this study has a clear focus on exploring and clarifying the expectation and acceptability of smart technologies in nursing home settings for relatively healthy elderly people. We made this clearer in the revised manuscript, please see line 110, page 5. |
| **Comment 2** | **2. I do not get the point of presenting the section on theoretical framework. The factors that are proposed in Figure 1. are different from what is explained in this section. I do not find factors such as self-efficacy or openness of information in Figure 1. Moreover, how do you separate the expectation and the confirmation part of the theoretical model? Expectation is pre-usage phenomenon, while confirmation is the post-usage scenario. How do you handle this aspect in your model? There are no hypothesis and I am unsure what the authors are trying to claim. You must be having proper hypotheses for measuring the user perception towards smart technology, else I doubt what really is the objective and the outcome of this work. In short, what I mean is the theoretical framework section needs a major overhaul with proper hypotheses keeping in mind the research objectives.** |
| Reply | 2-1: Apologies for the confusion. We have improved on theoretical framework diagram on figure 1.  2-2: The expectation and acceptability in the theoretical model are not two separate constructs but rather expectation is a determinant of acceptance if the smart nursing home appraisal meets the expectation. The acceptance expressed by the respondents in the study is not a confirmation but rather a precursor to the actualisation of the intention (confirmed acceptance) because the study does not capture or follow-up the respondents until their adoption of a smart nursing home. Therefore, the study hypothesis is that having an expectation of a smart nursing home is associated with better resilience to external and internal changes in appraisal of needs, and increases the acceptance of the smart nursing home. We made this clearer in the revised manuscript, see the section of ‘Theoretical framework’ on page 6-7 and the research objectives on page 8. |
| **Comment 3** | **3. In the study aims and objective section the authors state that “The aims of this study are to explore the expectations and acceptability of the mainland Chinese elderly people to smart nursing homes, and to determine the levels of these expectations and acceptability, as well as the sociodemographic factors associated with this matter”. The objective clearly points out that the authors are focusing on two distinct and theoretically different aspects of expectation and confirmation. So, please elaborate how to do so? Moreover, how do define and determine the different levels of expectation and acceptability? How many different levels do you have and how do you come up with these different levels? The information provided in this section is too vague and you must add more details. Second, smart nursing homes and smart homes are the same? If not, how they are different. I mentioned this point before also. How will be the perception of elderly people be different if focusing on smart nursing homes vs. smart homes? If you are trying to focus on various independent assistant living technologies (for otherwise healthy elderly people) for improving the quality of life, in that case I doubt the selection of nursing homes will not be a proper choice. So, please clarify on this aspect.** |
| Reply | 3-1: We have clarified the study hypothesis, questions and objectives. These will be achieved over three phases. In Phase I: the qualitative study, the expectation and acceptability of a smart nursing home will be explored through interviews. In Phase II, a questionnaire will be developed based on the findings of the qualitative study. Please see the first paragraph of ‘Methods’ on page 9 and figure 2 for the proposed plan for this process. In Phase III, the quantitative study, 5-point Likert score will be used to measure the scales of expectation and acceptability, and this will determine the level of expectations and acceptability.  3-2: Smart nursing home differs from a smart home. Smart nursing home could be an intelligent nursing care for the elderly people who reside at an institutional care facility. ‘Ageing in place’ is desirable, however, a number of elderly people may require a transition from home-based care to institutional care with a 24-hour supervision due to changes in health status and non-availability of family members who are able to care for them at home. These elderly people also perceive a nursing home that is easily accessible by others could offer an alternative living environment when experiencing a decline in health. |
| **Comment 4** | **4. I am not sure about Objective #3. If you are basing this work on some already existing theoretical model (as shown in Figure 1), then why do you need to propose a new scale? In fact, the constructs that are presented in Figure 1 are not novel, and they have been used in numerous contexts over time. So, what is the purpose of developing a new scale, when you already have pre-existing ones? Similarly, objectives #4 and #5 lack clarity. As I mentioned before what do you mean by different levels? How do you differentiate between the different levels? What is your theoretical justification between the said different levels? There are just too many unknowns. Similarly, what are the different categories of expectation and how do you obtain them?** |
| Reply | 4-1: Objective 3 is the transition and conversion of the mixed methods study design from the qualitative research strategy to quantitative by producing a conceptually and culturally valid scale (questionnaire) to measure the identified constructs of expectation and acceptability of smart nursing homes. It is guided but not confined by the theoretical model. The new scale that is developed from the Phase I qualitative approach ensures it of high content and construct validity that may not be obtained by adopting scales from different cultures, settings and time.  4-2: A 5-point Likert score will be used in Phase III to measure the scales of expectation and acceptability, and this will determine the level of expectations and acceptability. From the scores to the items or summed scores to the items, we may categorise the expectation and acceptability into 2 or 3 categories by combining the lower and upper responses into separate categories. This may be done at the item level or at each of the construct level depending on the dimensionality and psychometric property of the scale.We improved the clarity of this in the revised manuscript, please see line 342, paragraph 2, page 14. |
| **Comment 5** | **5. I am not sure of the rationale behind selecting the two specific cities for the purpose of the interview. What are the specialties of these cities and how can you ensure that these cities are representative of China in general?** |
| Reply | The ‘migratory birds elderly people’ in these two cities fly in from more than 20 provinces across China and they are expected to be of various psychosocial and demographic background of the larger elderly population in China who are the prospective users of smart nursing homes. These two cities are chosen for the Phase I qualitative study which aims to recruit variety of the elderly people to provide rich qualitative data and achieving the representativeness of the larger elderly population in China is the aim of the Phase III nationwide survey. Therefore, the mixed methods design of this study is considered most appropriate in exploring and quantifying the expectations and acceptability to a smart nursing home. |
| **Comment 6** | **6. Who will be the subjects for the semi-structured and the in-depth interviews? Are they going to be the same/different? Why/why not? How do you pre-determine the sample size to be around 50? What logic do you base this decision on? For example, the sample size has a high correlation to the number of questions that you are going to ask in the survey. Therefore, how do you select these numbers and ensure that it is optimum? The proposed interview durations are too long.** |
| Reply | 6-1: The eligibility criteria of participants for semi-structured interviews and in-depth interviews are the same, see our reply above. In-depth interviews may be conducted with certain respondents who have attended the previous interviews to gain more perspectives and to clarify the findings from the focus groups. Please see the revision in line 287, paragraph 2, page 12.  6-2: We have revised the sample size for qualitative research and the final size will be determined when a point of data saturation is reached. Please see line 301, page 13, ‘We will recruit 30 participants first, but the final sample size for this qualitative study will be determined when a point of data saturation is reached, for example, when additional data collection reveals no substantial new information.  6-3: We have revised the duration of interviews as ‘The duration of the individual interview will take between 30 and 45 minutes and the group interview will take around 45 to 60 minutes’. Please see line 303, page 13. |
| **Comment 7** | **7. The proposed methodology and contributions are not novel in my opinion, and there are already a multitude of studies on this aspect.** |
| Reply | Allow us to share on the current situation on what is known from the existing literature and available evidence on smart nursing homes. Kindly see our reply to the general comment 7 as well as the comment 1 from reviewer 1. We would like to highlight that this proposed study is supported by a preceding scoping review that will generate a clear concept of smart nursing home. This concept is guided by established theoretical framework, and further contextualized to Chinese elderly people using the sequential explanatory mixed methods study designs. We believe it is a sufficiently robust approach to the important research question of knowing the expectation and acceptability of a smart nursing home to the elderly people and their family members. To our best knowledge, there is no if any few studies that have all these features. Although this study is undertaken in only one country with its peculiar cultures and government policies, we believe the formation of the smart nursing home model is potentially applicable to other developed and developing countries. |

| **Comments of Reviewer 2:**  Reviewer #2: Thank you for the research work and review report. The paper tries to focus on Smart Nursing home model with some recent work their advantages and disadvantages. There are few points that must be addressed | |
| --- | --- |
| **Comment 1** | **1. Paper does not have the meaningful and significant abstract.** |
| Reply | We have revised the abstract of this mixed methods study protocol. |
| **Comment 2** | **2. Research does not include recent Chinese Smart Home care work**. |
| Reply | Please see the above reply to the ‘general comments 7’ and line 110, page 5 in manuscript. We would appreciate to be informed if they are other related ‘recent Chinese Smart Home care work’ to this study. |
| **Comment 3** | **3. The review is done very brief it does not cover the topic completely, it should be extended.** |
| Reply | We have improved on the background by providing more information, please see the section of ‘introduction’. |
| **Comment 4** | **4. There are some lengthy sentences and complex phrases, these should be addressed to improve the readability.** |
| Reply | We have improved accordingly and a proofreading was also done to the revised manuscript. |
