# Supplementary material for: The expectations and acceptability of a smart nursing home model among Chinese elderly people: A mixed methods study protocol
Source: PLoS One. 2021 Aug 23;16(8):e0255865. doi: 10.1371/journal.pone.0255865 (PMC8382194; doi:10.1371/journal.pone.0255865)
Supplement: Attachment — Submitted filename: Response to Reviewers-PONE-D-20-23914R2.docx [file pone.0255865.s004.docx]

| To PLOS ONE  Replies to comments to the authors  Manuscript ID: PONE-D-20-23914R2  Title of Manuscript: The Chinese elderly people’s expectation s and acceptability to a smart nursing home model: A mixed methods study protocol  Replies to the comments of the reviewer #2  Dear Reviewers,  We thank you for your valuable comments and we have revised the manuscript accordingly. Below, please find our response to your comments. | |
| --- | --- |
| Reviewer #2: Thank you for the research on elderly care and nursing. Please address the comments below: 1. The research need to describe in detail how these protocol could solve the low adaptability of elderly nursing care 2. Please make a comparative analysis with other available elderly care protocol with the proposed one. 3. Describe the controlled environment context in detail where it could be implemented 4. Revise the abstract and conclusion to improve the readability. | |
| **Comment 1** | **The research need to describe in detail how these protocol could solve the low adaptability of elderly nursing care** |
|  | The findings from this study could offer the needed solution for the existing nursing homes to adapt and transform into ones that are acceptable to the nursing home stakeholders, the elderly people and their family members. We have included the suggested description. Please see lines 573-577: “Smart nursing homes will provide the elderly people an alternative solution with independent, safe and comfortable environment to home-based care. It could also be the solution to the existing nursing homes to adapt and transform to a tech-assisted nursing home operation and living environment that are welcomed by the elderly people and their family members.” |
| **Comment 2** | **Please make a comparative analysis with other available elderly care protocol with the proposed one.** |
|  | Thank you for the suggestion. Since we did not find other similar study protocols to our proposed study, we will be looking out for similar elderly care protocol or nursing home models throughout the period of the study, and will conduct comparison to them after completion of this study. Please see the mentioned context in lines 614 and 615.: “We will compare and discuss the results of this study to other similar studies if they are conducted elsewhere, reported and published.” |
| **Comment 3** | **Describe the controlled environment context in detail where it could be implemented** |
|  | If this comment refers to the setting where the study will be conducted, we would like to refer to the Setting section in the study protocol at lines 423-437.  If the comment is about implementation of a smart nursing home model, it is not the study’s objective to implement a smart nursing concept although it may be able to configure one or some based on the collected data (combined with our scoping review). We do have a plan to share the smart nursing home models that come out from this study, and may conduct another study to investigate the effects of smart nursing homes compared to a control group of nursing homes on important outcomes. Until then, we will not be able to describe the control group or environment context where the smart home concepts would be implemented. |
| **Comment 4** | **Revise the abstract and conclusion to improve the readability.** |
|  | We have revised these sections for the readability. Please see the sections of abstract (lines 26-45) and discussion (lines 568-621). |
